# Moderate intake of aspartame and sucralose with meals, but not fructose, does not exacerbate energy and glucose metabolism in estrogen-deficient rats

**DOI:** 10.3164/jcbn.19-15

**Published:** 2019-09-11

**Authors:** Jin Ah Ryuk, Suna Kang, James W. Daily, Byoung-Seob Ko, Sunmin Park

**Affiliations:** 1 Korean Medicine Convergence Research Division, Korea Institute of Oriental Medicine, Daejeon, 305-811, South Korea; 2 Department of Food and Nutrition, Obesity/Diabetes Research Center, Hoseo University, 165 Sechul-Ri, BaeBang-Yup, Asan-Si, ChungNam-Do, 336-795, South Korea; 3 Department of R&D, Daily Manufacturing Inc., Rockwell, NC, USA

**Keywords:** non-nutritive sweeteners, fructose, GLP-1, glucose, insulin signaling

## Abstract

Both nutritive and non-nutritive sweeteners may influence energy and glucose metabolism differently. The hypothesis that sucrose, fructose, aspartame, and sucralose intake differently modulate energy and glucose metabolism was tested in an estrogen-deficient animal model. At 30 min after giving aspartame and sucralose (10 mg/kg body weight), an oral glucose tolerance test (OGTT) was conducted with glucose, sucrose, and fructose in ovariectomized (OVX) rats. After OGTT, they were continuously fed high fat diets including either 10% corn starch (Control), 10% sucrose (Sucrose), 10% fructose (Fructose), 0.05% aspartame + 9.95% starch (Aspartame) or 0.05% sucralose + 9.95% starch (Sucralose) for 8 week. During 30 min after acute administration of aspartame and sucralose, serum glucose concentrations increased despite slightly increased serum insulin levels before glucose infusion. However, glucose tolerance was not significantly different among the groups. In chronic study, serum glucose concentrations were lowest and insulin highest at the overnight-fasted state in Aspartame and Sucralose. Postprandial serum glucagon-like peptide-1 (GLP-1) and insulin levels were higher in Aspartame and Sucralose than Control. Hepatic insulin signaling (pAkt → pGSK-3β) and phosphoenolpyruvate carboxykinase (PEPCK) expression were lower in Sucralose and Aspartame than the Fructose. Serum acetate levels produced by gut microbiota were higher were lower in the fructose group than Aspartame and Sucralose groups. In conclusion, aspartame and sucralose with a meal might be preferable sweeteners to fructose and sucrose in estrogen deficient rats, and possibly post-menopausal women; however, this needs to be confirmed in human studies.

## Introduction

Obesity increases the development of metabolic diseases such as type 2 diabetes, dyslipidemia, hypertension and stroke,^(1)^ and is associated with increased adipokines.^(1)^ After menopause, women develop central obesity which increases their susceptibility to metabolic diseases. Obesity is a major problem for menopausal women, but Asians are not as obese as Americans and Europeans.^(2)^ Asians easily develop hyperglycemia when insulin resistance increases due to obesity, since Asians have lower insulin secretion capacity than Caucasians.^(3)^ Consequently, Asians with the same body mass index (BMI) have a higher prevalence of metabolic diseases, especially type 2 diabetes, as Americans and Europeans.^(2,4)^ Because Asians have lower thresholds of BMI and body fat mass for obesity related pathologies, obesity is defined at a lower BMI in many Asian countries.

Average total sucrose intake has increased from 20.6 g in 1993 to 72.1 g in 2013, and average sucrose intake from processed foods increased from 10.1 g in 1993 to 44.7 g in 2013 in Korea. 34% of Koreans consume more than 50 g of sucrose daily from processed foods, which is a World Health Organization recommended limit. Among Korean children and adolescents, consumption of sucrose from sucrose-sweetened beverages was positively related to metabolic syndrome in the Korean Child-Adolescent Cohort Study.^(5)^ Moreover, the intakes of sucrose-sweetened and non-nutritive sweetener-sweetened beverages increase the risk of type 2 diabetes by 43% and by 21%, respectively, compared to water intake, in post-menopausal women.^(6)^ Thus, the marked increase in consumption of nutritive and non-nutritive may be a factor in the increased incidence of diabetes.

Obesity is considered to be caused by an imbalance between energy consumption and expenditure, and sucrose-sweetened beverage intake is often blamed for obesity. However, after adjusting for energy intake and physical activity, an association between sucrose-sweetened beverage intake and obesity risk is inconsistent for children, adolescents, and adults. One prospective cohort study revealed a significant association between sucrose-sweetened beverage intake and obesity risk in children^(7)^ while another one showed no significant association between sucrose-sweetened beverage intake and BMI.^(8)^ A systematic review could not draw a conclusion about the causal relationship between sucrose-sweetened drinks intake and obesity in intervention, prospective and cross-sectional studies.^(9)^ Sucrose intake is also reported to be associated with insulin sensitivity and insulin secretion.^(10)^ A 12-week treatment of 30% sucrose consumption increased weight gain and deteriorated glucose tolerance without changing serum insulin levels in mice.^(11)^ High intakes of sucrose and fructose, accompanied with high energy intake, increased fat deposition in the liver, adipose tissues and skeletal muscle, and elevated insulin resistance.^(12–14)^ However, it remains controversial.^(12)^

Non-nutritive sweeteners have been used as substitutes for sucrose. Some non-nutritive sweeteners such as aspartame and sucralose have no calories but sweetness is much higher than sucrose. In previous large cohort studies, the intake of non-nutritive sweeteners decreased body weight and the risk of type 2 diabetes and coronary heart diseases.^(15)^ However, recent studies have demonstrated that non-nutritive sweeteners stimulate adipogenesis and suppress lipolysis,^(16)^ but they do not influence the secretion of satiety hormones glucagon-like peptide-1 (GLP-1) and peptide YY or ghrelin from the intestines, gastrointestinal satiety peptides.^(17)^ Thus, the effects of sucrose and non-nutritive sweeteners on energy and glucose metabolism remain uncertain.

Most studies on the effects of sucrose and non-nutritive sweeteners on energy and glucose metabolism have been conducted by providing beverages to young animals and adolescents, but only a few studies have provided non-nutritive sweeteners in the meal instead of sucrose. Furthermore, post-menopausal women develop obesity with glucose and lipid dysregulation, and they often have high in sugar intake.^(6,10)^ Therefore, we hypothesized that sucrose, fructose and non-nutritive sweeteners (aspartame and sucralose) may differently influence energy and glucose metabolism, and we tested the hypothesis and explored the mechanism in an estrogen-deficient animal model.

## Materials and Methods

### Animal care

Female Sprague–Dawley rats aged about 10 weeks (231 ± 20 g) were housed in individual stainless steel cages in a dedicated animal facility with temperature maintained at 23°C and a 12-h light/dark cycle. This study conformed with the Guide for the Care and Use of Laboratory Animals (8th edition, National Academies Press) and Use of Laboratory Animals and was approved by the Hoseo University Animal Care and Use Committee (HTRC-16-15). The rats obtained from DBL (Yeumsung-Kun, Korea) were allowed to acclimate for 1 week.

### Ovariectomy operation

After anesthetization by subcutaneously injecting ketamine and xylazine mixture (100 and 10 mg/kg body weight) the rats had ovariectomy (OVX) as previously described.^(18)^ The OVX rats were then randomly divided into 5 groups of 12 each.

### Experimental design

Fifty OVX rats were divided into 5 groups using a randomized block design. Each group was provided free access to water and an assigned diet (starch, sucrose, fructose, aspartame and sucralose). After 8 weeks of dietary intervention, the rats were fasted overnight and serum glucose concentrations were determined. Every week food intake was measured.

### Acute oral glucose tolerance test

Prior to providing assigned diets, oral glucose tolerance test (OGTT) was conducted by orally giving 2 g of different sweet molecules per kg body weight, or by administering aspartame and sucralose (10 mg/kg body weight) at doses with equivalent sweetness of 2 g glucose in OVX rats. Since sucrose and fructose themselves increase blood glucose levels, they were orally given instead of glucose. However, aspartame and sucralose do not increase blood glucose concentration itself although they may change serum insulin levels. At 30 min after oral intake of aspartame and sucralose the rats were administered 2 g glucose/kg body weight and serum glucose and insulin levels were monitored. The solution containing sucrose, fructose or glucose was orally provided to the rats at 0 min for the rats of the Sucrose, Fructose and Glucose groups. Every week 2 rats from each group had an OGTT and then had a wash-out period for 6 days. In the following week rats had another OGTT with another sweet molecule. 10 rats were included in each group of glucose, sucrose, fructose, aspartame + glucose and sucralose + glucose. Serum glucose concentrations were measured at −30, −15, 0 and every 10 min until 120 min and serum insulin concentrations were determined at −30, 0, 20, 40 and 90 min. Serum GLP-1 levels were assayed at 0 and 60 min by GLP-1 ELISA kits (Crystal Chem, Elk Grove Village, IL).

### Diet preparation

All groups were fed high fat diets [43 energy % (En%)] to exacerbate the energy, glucose and lipid metabolism after menopause as compared to diets low in fat.^(19–21)^ Diet composition was 37% from carbohydrate (10 En% sucrose and 27 En% starch), 20 En% from protein (casein) and 43 En% from lard which was based on the modified AIN-93 formulation.^(22)^ To determine the effects of non-nutritive sweeteners and natural sweeteners on energy and glucose metabolism in OVX rats, 10 En% sucrose in the original modified AIN-93 formulation was switched to starch (10 En% Control), fructose (10 En% Fructose), sucrose (10 En% Sucrose), starch (9.5 En%) plus aspartame (0.05 En% Aspartame), and starch (9.8 En%) plus sucralose (0.05 En% Sucralose). Since aspartame and sucralose have over 100-fold sweetness more than sucrose, they were used 200 times less than sucrose and the remaining portion was filled with starch.

### Energy expenditure by indirect calorimetry

At the 7th week of the assigned treatment, energy expenditure was assessed at the beginning of the dark phase of the light–dark cycle after 6 h of feed-deprivation. The rats were placed into metabolic chambers (airflow = 800 ml/min) equipped with a computer-controlled O_2_ and CO_2_ measurement system (Biopac Systems Inc., Goleta, CA). The respiratory quotient (RQ) and resting energy expenditure (REE) were calculated using the equations provided by Niwa *et al.*^(23)^ Average oxygen consumption (VO_2_) and average carbon dioxide production (VCO_2_) were calculated using previously published methods and used to calculate carbohydrate and fat oxidation and the amount of oxygen consumed per gram of substrate oxidized.^(24,25)^

### Glucose homeostasis and sample collection at the end of experiment

At the 7th week, blood was collected from overnight-fasted animals and at 30 min after assigned food intake. Serum was collected by centrifuging the blood at 3,000 *g* for 10 min and GLP-1 levels were measured by ELISA kit (Crystal Chem). At 2 days later the rats were subjected to an OGTT by oral administration of 2 g of glucose/kg body weight. At 10 min intervals from 0 to 120 min post glucose loading, tail blood was collected for serum glucose measurements using a Glucose Analyzer II (Beckman, Palo Alto, CA). At 0, 20, 40, 90, and 120 min serum insulin concentrations were assessed using a ultra-sensitive rat insulin ELISA kit (Crystal Chem). The trapezoidal rule was used for calculating means of the total area under the curve (AUC) for serum glucose and insulin concentrations.

Three days’ post OGTT, the rats were anesthetized with the ketamine/xylazine as used earlier in the study and epididymal and retroperitoneal fat masses and uteri were excised and weighed. The uterus index, was calculated as uterus weight divided by body weight. Blood was collected from the portal vein and inferior vena cava for measuring short-chain fatty acids and other metabolic samples, respectively. Serum was prepared from the blood by centrifuging at 3,000 rpm for 20 min. Human insulin (1 U/kg body weight) was then injected into the inferior vena cava for determining hepatic insulin signaling. Serum and tissues were then stored at –70°C for future use.

The homeostasis model assessment estimate (HOMA) for assessing insulin resistance (IR) and HOMA for insulin secretion (B) were calculated as previously reported.^(22)^ Serum 17β-estradiol levels were measured by ELISA kits (Enzo Life Sciences, NY). Serum triglyceride concentrations were measured by using colorimetry kits (Asan Pharmaceutical, Seoul, Korea).

### Short-chain fatty acid analysis by gas chromatography

The solution of *n*-butanol:tetrahydrofuran:acetonitrile (50:30:20, v:v:v) was mixed with serum with equal volume, and 5 M HCL was added into the mixed solution. The mixture was vortexed for 5 min and centrifuged at 4°C, 3,000 *g* for 15 min. The supernatant was taken and it was injected into Gas chromatography 680 (PerkinElmer Clarus, Waltham, MA) with Elite-FFAP column (30 m × 0.25 mm × 0.25 µm). The carrier gas was helium and the flow rate was 1 ml/min. The temperature was raised until 180°C at 10°C/min, and then the temperature was raised to 240°C at 20°C/min and retained for 6 min. The inlet and detector temperature were 230°C and 250°C, respectively. The flow rates of hydrogen, air, and helium were 45, 450, and 20 ml/min, respectively.

### Immunoblot analysis

Livers were lysed in 20 mM Tris buffer as previously reported.^(19)^ Lysates equilibrated to equal amounts of protein (30–50 µg) were immunoblotted with specific antibodies against protein kinase B (PKB/Akt), glycogen synthase kinase (GSK)-3β, phosphoenol-pyruvate carboxykinase (PEPCK), and β-actin, and phosphorylated forms of PKB^Ser473^ and glycogen syntase kinase-3β (GSK-3β) (Cell Signaling, Danvers, MA), as previously described.^(19)^ Intensities of protein expression were measured using Imagequant TL (Amersham Biosciences, Piscataway, NJ).

### Statistical analysis

SAS software version 7 (SAS Institute, Cary, CA) was used for statistical analysis. Sample size was estimated using a G power program (power = 0.90 and effect size = 0.5) and a sample size of 10 per group was required. When the results were normally distributed as confirmed by using Proc univariate, results are given as means ± SD. Variables spanning multiple time points were analyzed using two-way repeated measures analysis of variance (ANOVA), with independent variables being time and group and the interaction term being between time and group. Measurements were statistically analyzed by one-way ANOVA. Significance of differences among the multiple groups was assessed by Tukey’s test at the level of *p*<0.05.

## Results

### Acute OGTT

**Fig. 1:**
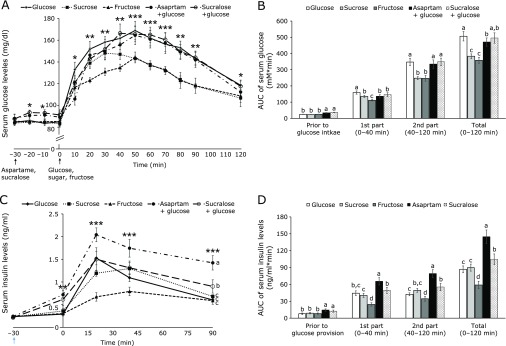
Serum glucose and insulin levels after oral infusion of glucose, sucrose or fructose prior to the oral intake of distilled water, aspartame, or sucralose. Rats had 10 mg aspartame, sucralose or distilled water per kg body weight and at 30 min later, the rats had 2 g glucose, fructose or sucrose per kg body weight. Rats in the Aspartame and Sucralose groups had glucose and those in the Sucrose and Fructose had sucrose and fructose, respectively. Serum glucose levels were measured every 10 min after giving aspartame, sucralose and distilled water (−30 min) (A). Area under the curve (AUC) of serum glucose levels was calculated in 3 parts (B): prior to oral glucose infusion (−30–0 min), 1st part after glucose infusion (0–40 min) and 2nd part after glucose infusion (40–120 min). Serum insulin levels were measured at −30, 0, 20, 40, and 90 min (C). AUC of serum insulin levels were assayed in the 1st part after glucose infusion (0–20 min) and 2nd part after glucose infusion (20–40 min) (D). The dots and bars represent means ± SD (*n* = 12). *Significantly different among all groups in one-way ANOVA at *p*<0.05, ** at *p*<0.01, *** at *p*<0.001. ^a^^,^^b^^,^^c^The different letters on the bars represent significant differences among the groups by Tukey’s test at *p*<0.05.

At 30 min after aspartame and sucralose administration, serum glucose concentrations had increased about 6–8 mg/dl compared to saline treatments in OVX rats. Since aspartame and sucralose do not include energy sources, 2 g glucose per kg body weight was orally given. Sucrose or fructose (2 g per kg body weight) was orally provided instead of glucose. Serum glucose levels increased until 30–40 min after glucose, sucrose or fructose provision and the levels were lowered after the peak (Fig. 1A). The peak levels were lowest in the fructose group among the groups and the levels were lower in sucrose group in the second part of the OGTT (Fig. 1A). The concentrations were similar in glucose administered group with or without aspartame and sucralose uptake. After the peak serum glucose levels were lowered in all groups. Sucrose quickly decreased serum glucose levels than the glucose and glucose plus aspartame or sucralose. Aspartame and sucralose consumption did not affect serum glucose levels in the 2nd part of OGTT (Fig. 1B). Area under the curve (AUC) during entire OGTT was highest in the Glucose group among all groups and the AUC was lower in the Aspartame group than the Glucose slight but significantly (*p* = 0.048). Sucrose and Fructose groups were lower than the Aspartame group (Fig. 1B).

Serum insulin levels were slightly elevated at 30 min after aspartame and sucralose were given in comparison to the saline groups in OVX rats (Fig. 1C). Glucose or sucrose provision increased serum insulin levels at 20 min and then the levels were lowered. Glucose with aspartame increased serum insulin levels the most at 20 min among the groups. However, fructose increased serum insulin levels much less than other groups at 20 min, but remained elevated until at least 45 min (Fig. 1C). Sucrose was digested into glucose and fructose and the serum insulin levels were in between of glucose and fructose administration (Fig. 1C). Even though serum glucose levels in the Aspartame and Sucralose groups were similar to the Glucose group, serum insulin levels were much higher in the Aspartame and Sucralose groups during entire OGTT (Fig. 1C). Serum insulin levels were highest in the Aspartame among the groups.

AUC of serum insulin concentrations were higher in the aspartame and sucralose groups than the other groups for 30 min after aspartame and sucralose intake and remained elevated in the Aspartame group 30 min longer than in the sucralose group (Fig. 1D). After 2 g/kg body weight glucose, fructose or sucrose intake AUC in the 1st part was higher in the ascending order of fructose, sucrose, glucose, sucralose and aspartame. The AUC in the 2nd part also showed a similar pattern to that of the 1st part (Fig. 1D). AUC of serum insulin during entire OGTT were elevated with aspartame and sucralose administration with glucose compared to glucose administration only (Fig. 1D). Fructose administration lowered the AUC of serum insulin levels than glucose administration (Fig. 1D). The results indicated that in acute administration of artificial sweetener and glucose, aspartame and sucralose treatment increased insulin resistance and aspartame elevated it more than Sucralose during OGTT.

### Energy metabolism and short-chain fatty acids in the circulation

Final body weight was not significantly different among the different interventions. Weight gain during experimental periods tended to be higher in the Fructose group than the other groups but it was not significantly different (*p* = 0.07; Table 1). However, uterine fat mass was much higher in the Fructose group than in the other groups whereas retroperitoneal fat mass was lowered in the descending order of Fructose, Sucrose, Control, Aspartame and Sucralose (Table 1). Visceral fat mass, the sum of uterine fat and retroperitoneal fat mass, was highest in the Fructose group among all groups and it was higher in the Sucrose and Control groups than the Aspartame and Sucralose groups (Table 1). Uterus weight and serum 17β-estradiol levels were not significantly different among all the groups (Table 1).

Among short-chain fatty acids serum acetate levels were higher only in the Fructose compared to the Control (Table 1). By contrast, serum propionate and butyrate levels were not significantly different among the groups. They tended to be lower in the Fructose than the Control (Table 1).

Food intake was not significantly different among the groups (Table 2). Energy expenditure was lower in the Fructose group than in the Control group (Table 2). Oxygen consumption was lower in the Fructose group than the other groups. RQ was not significantly different among the groups. Carbohydrate oxidation was lowered in the descending order of Aspartame, Sucralose, Control, Sucrose, and Fructose (Table 2). Fat oxidation was opposite to carbohydrate oxidation (Table 2). Thus, the greater weight gain in the Fructose group was associated with lower energy expenditure, not higher energy intake. Aspartame and sucralose did not alter carbohydrate and fat oxidation compared to the Control (Table 2).

### Glucose metabolism

Serum glucose concentrations at the overnight fasting state decreased in the descending order of the Fructose, Sucrose, Aspartame, Control and Sucralose (Table 3). Serum insulin concentrations at the overnight fasted state showed an opposite trend to serum glucose concentrations. Serum insulin levels were lowest in the Fructose group and the levels were highest in the Aspartame group among the groups (Table 3). HOMA-IR, an index of insulin resistance, was highest in Sucrose and Aspartame groups among the groups and it was higher in the Fructose and Sucralose than the Control. HOMA for insulin secretion (HOMA-B), an index of insulin secretion, was elevated in the ascending order of Aspartame = Sucralose, Control, Sucrose, and Fructose (Table 3). Thus, sucalose might be preferable to fructose and sucrose in glucose metabolism.

**Fig. 2:**
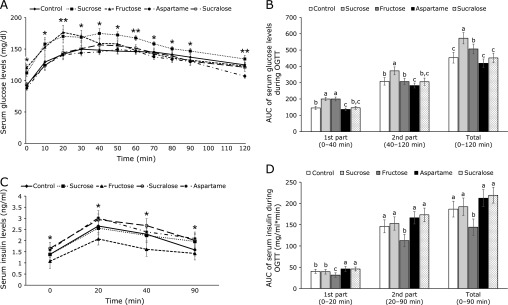
The changes of serum glucose levels and areas under the curve of glucose and insulin during the oral glucose tolerance test (OGTT) after 7 week consumption of the assigned sweeteners. The ovariectomized (OVX) rats were provided with a 45% fat diet with 10% starch, sucrose, fructose, aspartame + starch and sucralose + starch for 8 weeks. At the 7th week, 2 g of glucose/kg body weight was orally administered and the serum glucose and insulin levels were measured at the indicated times. The changes in the serum glucose (A) and insulin (B) levels were measured during the OGTT. The average of the area under the curve (AUC) of glucose (C) and insulin (D) during the first part (0–40 min) and second part (40–120 min) of the OGTT. The dots and bars represent means ± SD (*n* = 12). *Significantly different among all groups in one-way ANOVA at *p*<0.05, ** at *p*<0.01. ^a^^,^^b^^,^^c^The different letters on the bars represent significant differences among the groups by Tukey’s test at *p*<0.05.

After the glucose challenge, serum glucose concentrations in all groups increased until 20–40 min and then they gradually decreased (Fig. 2A). The peak levels were markedly higher in the Fructose and Sucrose groups than the other groups (Fig. 2A). Serum glucose concentrations markedly decreased after the peak at 20 min in the Fructose group but the concentrations in the Sucrose group were maintained at the peak levels until 50 min. Serum glucose concentrations at the peak were much lower in the Control, Aspartame and Sucralose groups than the Sucrose and Fructose groups (Fig. 2A).

The AUC of serum glucose levels at the 1st part was much higher in the Sucrose and Fructose groups than the other groups whereas the AUC in the Aspartame group was lower than the Control (Fig. 2B). The AUC of serum glucose levels at the 2nd part was biggest in the Sucrose group among all the groups and that was smallest in the Aspartame group (Fig. 2B).

The changes in serum glucose concentrations during OGTT were associated with insulin secretion and insulin resistance. Serum insulin concentrations were elevated until 20 min and decreased during OGTT in the rats of all groups (Fig. 2C). The peak serum insulin levels at 20 min were higher in the ascending order of the Fructose, Sucrose and Control, Aspartame, and Sucralose groups (Fig. 2C). Serum insulin concentrations declined after the peak levels in different patterns. The AUC of serum insulin concentrations at the 1st part (0–20 min) during OGTT was lowest in the Fructose group and it was higher in the non-nutritive sweetener groups than the Control and Sucrose groups (Fig. 2D). The AUC of the 2nd part (20–90 min) was lowest in Fructose among the groups and the AUC was not significantly different among the other groups (Fig. 2D). These results indicated that insulin resistance during hyperglycemia was higher in the Sucralose than the Fructose group.

**Fig. 3:**
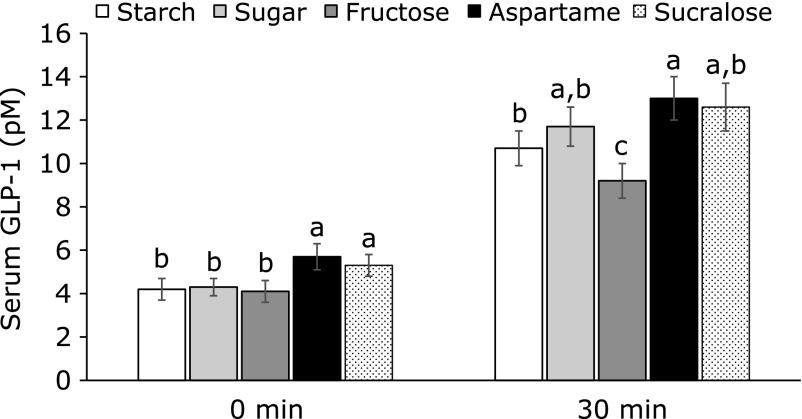
Serum GLP-1 levels at 30 min after assigned meal intake. After 7 weeks of feeding with a 45% fat diet with 10% starch, sucrose, fructose, aspartame + starch and sucralose + starch in the ovariectomized (OVX) rats, the rats had blood collection in overnight fasting state and 1 h food provision. Serum GLP-1 levels were measured from the blood. The bars represent means ± SD (*n* = 12). ^a^^,^^b^^,^^c^The different letters on the bars represent significant differences among the groups by Tukey’s test at *p*<0.05.

After providing food for 30 min, serum GLP-1 levels were higher in the Aspartame and Sucralose groups than the other groups at 0 min (Fig. 3). Serum GLP-1 levels at 30 min were higher in the Aspartame group than the Control group and the levels were lowest in the Fructose group among the groups (Fig. 3). Serum triglyceride levels were higher in the Fructose than the Control and Sucralose and Aspartame lowered serum triglyceride concentrations below that of the control (Table 3).

### Hepatic insulin signaling

**Fig. 4:**
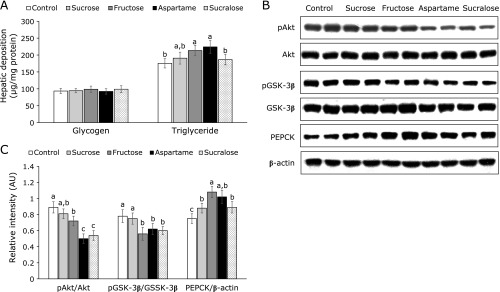
Hepatic insulin signaling at the end of experiment. The ovariectomized (OVX) rats were provided with a 45% fat diet with 10% starch, sucrose, fructose, aspartame + starch and sucralose + starch for 8 weeks. After 16 h fasting, regular human insulin (5 U/kg body weight) was injected through their inferior vena cava. Hepatic deposition of glycogen and triglyceride was measured (A). The hepatic insulin signaling were measured with immunoblotting (B). The band intensity was measured with image analyzer (C). The bars represent means ± SD (*n* = 6). ^a^^,^^b^^,^^c^The different letters on the bars represent significant differences among the groups by Tukey’s test at *p*<0.05.

Glycogen deposition in the liver was not significantly different among the groups (Fig. 4A). However, triglyceride accumulation in the liver was greater in the Aspartame group than the Control group (Fig. 4A).

In the liver the phosphorylation of Akt was lower in the Aspartame and Sucralose groups than the other groups and it was lowered by Fructose to less than the Control (Fig. 4B). Consistent with phosphorylation of Akt, the phosphorylation of GSK-3β also decreased in the Fructose, Aspartame and Sucralose groups more than in the Control (Fig. 4B). However, PEPCK expression increased in aspartame and Fructose group compared to the Control group (Fig. 4B). Aspartame and Sucralose were higher than the Control, but not Fructose group. Thus, other signaling influenced PEPCK expression other than insulin signaling to increase hepatic glucose output in the Fructose group.

## Discussion

Aspartame and sucralose are most commonly used in processed foods, but they have some potential to have harmful effects on energy and glucose metabolism although non-nutritive sweeteners contribute little or no energy to the foods despite their sweetness.^(26,27)^ However, the results of metabolic studies are inconsistent, and their mechanisms are complicated. This study evaluated the acute and chronic effects of administering glucose, sucrose, fructose, aspartame or sucralose on energy metabolism, especially glucose metabolism and insulin secretion. In the acute OGTT rats administered aspartame, and to a lesser extent sucralose, had much higher peaks and AUC of insulin concentrations, even though glucose concentrations were almost the same as other groups receiving same doses of glucose. This suggested that aspartame and sucralose acutely interfered with insulin action and much more was required to normalize blood glucose concentrations. Long-term administration aspartame and sucralose caused the rats to require somewhat higher insulin levels to normalize blood glucose concentrations in OGTT without their simultaneous administration. Both aspartame and sucralose bind to a similar region of the sweet receptor.^(28)^ The present study demonstrated that rats fed non-nutritive sweeteners (aspartame and sucralose) were more insulin resistant than the control in an estrogen-deficient animal model, even though they had less visceral fat mass. It was associated with higher insulin secretion capacity in non-nutritive sweetener groups.

Glucose, fructose and sucrose increase insulin secretion not only by elevating serum glucose levels after their absorption but also by activating sweet taste receptor in the tongue, intestines, pancreatic β-cells and brain.^(29)^ The sweet taste receptors in the tongue act at the forefront of nutrient sensing to deliver the signal to prepare to maintain glucose homeostasis.^(30)^ Different sweeteners differently affect the taste receptor activation. Glucose triggers taste receptor signaling in the pancreatic β-cell to increase intracellular Ca^++^ that activates phospholipase and transient receptor potential cation channel, subfamily M, member 5 (TRPM5).^(31)^ The TRPM5 mediates glucose-stimulated insulin secretion by GLP-1.^(32)^ The common TRPM5 variants are associated with prediabetic phenotypes, and they may act as risk factors for type 2 diabetes.^(33)^ Fructose does not activate TRPM5, but it specifically activates taste receptor type 1 member 2 (T1R2) on pancreatic β-cells, and fructose has synergistic activity with glucose to amplify insulin release in human and mouse islets.^(31)^ Moreover, non-nutritive sweeteners activate T1R2 receptors in the pancreatic β-cells. Human studies demonstrate that the consumption of an oral solution of sucrose (disaccharide of glucose and fructose) can potentiate insulin release in comparison to the equimolar amounts of glucose alone, but the intake of non-nutritive sweeteners fail to modulate serum insulin or glucose levels.^(34,35)^ In the present study nutritive and non-nutritive sweeteners increased glucose-stimulated insulin secretion in estrogen-deficient rats, possibly by activating taste receptors in the organs.

Fructose is passively but rapidly absorbed in the intestines through glucose transporter 5 (GLUT5) and it is delivered into the liver via the portal vein.^(36)^ Fructose has a much lower glycemic index than glucose indicating that fructose does not increase insulin secretion as much as glucose. Fructose increases fatty acid synthesis without regulating glycolysis, and it increases the synthesis of triglycerides, which are deposited in the liver; hepatic oxidative stress and inflammation were also elevated which induce hepatic steatosis.^(37)^ Furthermore, the present study also showed triglyceride deposition was higher in the Fructose group than in the Control. It did not induce non-alcohol liver steatosis since the rats had a relatively low fructose diet (11%). The fructose diet increased glycogen accumulation in OVX rats in the present study, which is possibly related to the production of fructose-1 phosphate from fructose in the liver since fructose-1-phosphate is a known inhibitor of glycogen phosphorylase that is involved in the degradation of glycogen. It might also increase gluconeogenesis from amino acids from the muscle and increased deposition of glycogen and lipid might induce hypertrophy of the liver.

In addition, increased serum acetate levels produced by gut microbiota have reported to be associated with obesity and insulin resistance by stimulating insulin secretion with activating parasympathetic nervous system.^(38)^ In the present study fructose intake increased visceral fat mass and insulin resistance with elevating serum acetate levels, indicating that high fructose in the diet increased production of acetate by gut microbiota. However, increased serum acetate levels did not increase insulin secretion in the Fructose group although serum glucose levels were much higher in the Fructose than other groups. As a result, fructose might suppress serum insulin levels in another way. By contrast, aspartame and sucralose did not alter serum acetate levels although they increased serum insulin levels. Therefore, high intake of fructose may facilitate the development non-alcoholic hepatic steatosis, obesity and type 2 diabetes, even though fructose has a lower glycemic index. A moderate intake of aspartame and sucralose may not exacerbate energy and glucose metabolic and gut microbiome.

Chronic intake of aspartame and sucralose also influence glucose metabolism,^(39,40)^ although the effect is more moderate that seen with acute administration. Non-nutritive sweeteners such as sucralose and aspartame stimulate taste receptors to signal the brain to communicate the need for pancreatic β-cell cells to prepare for insulin and GLP-1 secretion.^(40)^ Non-nutritive sweeteners stimulate the T1R2-T1R3 in the pancreatic β-cells to release insulin. Interestingly, chronic consumption of aspartame and sucralose increased serum insulin levels more than the control diet during OGTT. At 30 min serum GLP-1 levels were higher in the Aspartame and Sucralose groups than in the Control in the present study. Thus, sweet taste itself increased GLP-1 secretion to promote insulin. Sylvetsky *et al.*^(41)^ showed that non-nutritive sweeteners increase serum insulin concentrations and also GLP-1 secretion. Only diet soda containing aspartame elevates GLP-1 response in human subjects.^(41)^ Non-nutritive sweeteners may elevate GLP-1 response to release insulin from the pancreatic β-cells, but no glucose enters into the blood circulation. However, the secretion of GLP-1 and gastric inhibitory polypeptide by non-nutritive sweeteners is still controversial.^(42,43)^ In our acute administration of aspartame and sucralose both slightly increased glucose and insulin levels without glucose intake and in their chronic administration they elevated HOMA-IR. This could be a compensatory mechanism to protect against hypoglycemia in response to exaggerated insulin release when little or no glucose is absorbed into the blood. Increased HOMA-IR might be associated with the half-life of non-nutritive sweeteners. Saccharine and sucralose are about 13–14 h but acesulfame K and aspartame are very short.^(41)^ Saccharine and sucralose effect on insulin and GLP-1 secretion may be sustained for long periods. Therefore, consuming saccharine and sucralose in the fasting state may result in higher postprandial GLP-1 and insulin secretion when a meal is consumed much later, suggesting that the dosage and timing non-nutritive sweetener consumption may be important and needs to be studied in the future.

In conclusions, the chronic moderate intake of aspartame and sucralose instead of sucrose with meals may not exert harmful effects on glucose and energy metabolism in post-menopausal women, but even moderate intake of fructose intake may be detrimental to energy metabolism. Therefore, if the results of this animal model can be confirmed to be applicable to post-menopausal women, small amounts of aspartame and sucralose (less than 100 mg/day) with meals might may be preferable to sweeteners such as fructose and sucrose. Even though the non-nutritive sweeteners increased insulin resistance compared to the Control. Since sucralose and aspartame have different characteristics for energy, lipid and glucose metabolism, the mixture of sucralose and aspartame should be examined to make better non-nutritive sweetener combination for the processed foods. However, caution must be observed in recommending non-nutritive sweeteners based on animal data such as these, and although we did not find the artificial sweeteners to have severe metabolic effects in this animal model, humans may react differently. Furthermore, a recent study found that the use of beverages using artificial sweeteners greatly increased the risk of stroke in women.^(44)^ Therefore, based on our results and those of others, the best advice would be for people to avoid the use of added sweeteners as much as possible and to use glucose when a sweetener is necessary. However, human randomized clinical study is necessary to be confirmed.

## Author Contributions

Authors’ roles: Financial support: SP and BSK Study design: SP and JWD. Study conduct: JR, SK. Data collection: SK. Data analysis: SP, SK. Data interpretation: JR, SP, BSK, JWD. Drafting manuscript: SP. Revising manuscript content: JWD, JR, BSK. Responsibility for the integrity of the data analysis: JR and SP. Approving final version of manuscript: all authors.

## Figures and Tables

**Table 1:** Body weight, visceral fat mass and uterine weight

	Control (*n* = 12)	Sucrose (*n* = 12)	Fructose (*n* = 12)	Aspartame (*n* = 12)	Sucralose (*n* = 12)
Final body weigt (g)	301 ± 25	300 ± 26	309 ± 27	303 ± 21	303 ± 19
Body weight gain (g/8 weeks)	102 ± 9.3	102 ± 8.8	111 ± 10.7	105 ± 9.2	101 ± 9.7
Uterine fat mass (g)	4.70 ± 0.87^b^	5.04 ± 0.51^b^	7.31 ± 0.97^a^	4.64 ± 0.65^b^	4.94 ± 0.68^b^
Retroperitoneal fat mass (g)	3.78 ± 0.76^b^	4.24 ± 0.65^b^	6.16 ± 0.98^a^	3.29 ± 0.54^c^	3.20 ± 0.52^c^
Visceral fat mass (g)	8.6 ± 1.7^c^	10.6 ± 1.9^b^	13.5 ± 2.0^a^	7.7 ± 1.0^c^	8.1 ± 1.1^c^
Uterus weight (g)	0.25 ± 0.05	0.24 ± 0.06	0.25 ± 0.05	0.26 ± 0.05	0.25 ± 0.06
Serum acetate (µM)	165 ± 18^a^	167 ± 20^a^	192 ± 21^b^	172 ± 19^a^	169 ± 19^a^
Serum propionate (µM)	54.6 ± 5.7	53.1 ± 4.8	51.4 ± 5.9	55.5 ± 5.2	53.9 ± 5.6
Serum butyrate (µM)	24.6 ± 2.0	23.5 ± 1.7	22.7 ± 2.1	23.2 ± 1.9	23.6 ± 1.8

The ovariectomized (OVX) rats were provided with a 45% fat diet with 10% starch, sucrose, fructose, aspartame + starch and sucralose + starch in a respective group for 8 weeks. Visceral fat mass was calculated by sum of uterine fat mass and retroperitoneal fat mass. ^a^^,^^b^^,^^c^The different letters in the means represent significant differences among the groups by Tukey’s test at *p*<0.05.

**Table 2:** Energy metabolism

	Control (*n* = 12)	Sucrose (*n* = 12)	Fructose (*n* = 12)	Aspartame (*n* = 12)	Sucralose (*n* = 12)
Food intake (g/day)	15.1 ± 1.2	14.7 ± 1.2	14.5 ± 1.1	15.1 ± 1.2	15.8 ± 1.7
Energy expenditure (ml·kg^−0.75^·min^−1^)	126 ± 13^a^	119 ± 13^a,b^	107 ± 13^b^	131 ± 14^a^	129 ± 13^a^
VO_2_ (ml·kg^−0.75^·min^−1^)	17.7 ± 1.8^a^	17.0 ± 1.7^a,b^	15.3 ± 1.6^b^	18.4 ± 1.7^a^	18.4 ± 1.8^a^
Respiratory quotient	0.85 ± 0.07	0.85 ± 0.07	0.85 ± 0.08	0.86 ± 0.07	0.85 ± 0.08
Carbohydrate oxidation (mg·kg^−0.75^·min^−1^)	6.5 ± 0.7^a^	6.2 ± 0.7^a,b^	5.6 ± 0.6^b^	7.1 ± 0.8^a^	6.7 ± 0.7^a^
Fat oxidation (mg·kg^−0.75^·min^−1^)	6.9 ± 0.7^a^	6.5 ± 0.7^a,b^	5.8 ± 0.8^b^	6.6 ± 0.6^a^	7.0 ± 1.0^a^

The ovariectomized (OVX) rats were provided with a 45% fat diet with 10% starch, sucrose, fructose, aspartame + starch, and sucralose + starch in a respective group for 7 weeks. After 16 h fasting, energy expenditure was measured by indirect calorimeter. ^a^^,^^b^^,^^c^The different letters in the means represent significant differences among the groups by Tukey’s test at *p*<0.05.

**Table 3:** Glucose metabolism in overnight-fasted states

	Control (*n* = 12)	Sucrose (*n* = 12)	Fructose (*n* = 12)	Aspartame (*n* = 12)	Sucralose (*n* = 12)
Serum glucose (mg/dl)	89.1 ± 5.2^c^	109 ± 5.5^b^	118.2 ± 8.2^a^	90.0 ± 4.4^c^	86.7 ± 5.4^c^
Serum insulin (ng/ml)	1.22 ± 0.23^b,c^	1.29 ± 0.21^b^	1.07 ± 0.18^c^	1.54 ± 0.27^a^	1.43 ± 0.23^a,b^
HOMA-IR	6.0 ± 0.7^c^	7.8 ± 0.8^a^	7.0 ± 0.7^b^	7.7 ± 0.8^a^	6.9 ± 0.8^b^
HOMA-B	371 ± 43^b^	321 ± 45^c^	246 ± 29^d^	463 ± 46^a^	447 ± 54^a^
Serum triglyceride (mg/dl)	117 ± 13^b^	109 ± 8.6^b,c^	138 ± 14^a^	97.8 ± 5.9^c^	104 ± 6.7^c^

The ovariectomized (OVX) rats were provided with a 45% fat diet with 10% starch, sucrose, fructose, aspartame + starch, and sucralose + starch in a respective group for 8 weeks. HOMA-IR, homeostatic Model Assessment for Insulin Resistance; HOMA-B, HOMA for insulin secretion. ^a^^,^^b^^,^^c^The different letters in the means represent significant differences among the groups by Tukey’s test at *p*<0.05.

## Abbreviations

ANOVA: analysis of variance
AUC: area under the curve
BMI: body mass index
En%: energy percent
GLP-1: glucagon-like peptide-1
GLUT5: glucose transporter 5
GSK-3β: glycogen synthase kinase-3β
HOMA-IR: homeostasis model assessment estimate of insulin resistance
OGTT: oral glucose tolerance test
OVX: ovariectomy
PEPCK: phosphoenolpyruvate
REE: resting energy expenditure
RQ: respiratory quotient
T1R2: taste receptor type 1 member 2
VCO_2_: carbon dioxide production
VO_2_: oxygen consumption
